# HOME AND COMMUNITY-BASED SERVICE USE AMONG VETERANS WITH DEMENTIA VARIES BY COMORBIDITY LEVEL AND HEALTH CARE TEAM

**DOI:** 10.1093/geroni/igae098.2948

**Published:** 2024-12-31

**Authors:** Emma Quach, Lauren Moo, Emily Franzosa, Pengsheng Ni, Shibei Zhao, Christine Hartmann

**Affiliations:** VA Bedford Healthcare System, Bedford, Massachusetts, United States; Department of Veterans Affairs, Bedford, Massachusetts, United States; James J Peters VA Medical Center, Bronx, New York, United States; Boston University, Boston, Massachusetts, United States; Center for Healthcare Organization and Implementation Research, Bedford, Massachusetts, United States; VA Bedford Healthcare System & University of Massachusetts Lowell, Bedford, Massachusetts, United States

## Abstract

Home and community-based services (HCBS) such as home care and adult day centers (adult day) are vital to supporting adults with dementia in community settings. Using 2019 data of 143,281 patients with dementia from the Veterans Health Administration, we investigated HCBS use (use of both home care and adult day, use of only one service, and use of neither service) in older adults with dementia. These adults may receive care from one of three types of case-managed health care teams (Home Based Primary Care, geriatrics-based outpatient primary care, and dementia-focused outpatient specialty care). They had various comorbidity levels, measured as the number of (adapted) Elixhauser comorbidities index and dichotomized as < 4 or ≥ 4 comorbidities. We compared HCBS use (use of neither service as referent) by patients’ type of case-managed team (patients in no team as referent), stratified by patients’ non-dementia comorbidity levels (< 4 or ≥ 4). Use of both home care and adult day was associated with each type of health care team at each comorbidity level. Home Based Primary Care and dementia-focused specialty care were more consistently associated with HCBS use--only adult day and of only home care--than geriatrics-based primary care for patients with ≥ 4 non-dementia comorbidities. Our findings suggest that, in patients who have both dementia and other comorbidities, case-managed health care teams in primary and specialty care settings contribute to use of critical community supports. Among these, implementation of case management faced more challenges within outpatient primary care teams.

